# Mel Avery: Mentor, Role Model, Friend, Mother of Us all

**DOI:** 10.3389/fped.2014.00018

**Published:** 2014-03-31

**Authors:** Mary E. Sunday

**Affiliations:** ^1^Pathology, Pediatrics, Medicine, and Cell Biology, Duke University Medical Center, Durham, NC, USA

**Keywords:** twentieth century, historical perspective, neonatology, translational medical research, basic science research, mentoring

Dr. Mary Ellen Avery, affectionately known as “Mel,” was a woman of courage, with a sense of humor and deep humanity. She was an intellectual powerhouse, highly creative, and productive, similar to many leaders. Yet she became the most beloved mentor for many. Here, I share some personal memories hoping to inspire others juggling life and career responsibilities.

I first met Mel in 1977 when she taught our Harvard Medical School (HMS) class about respiratory distress syndrome (RDS). She held a critical audience entirely captive, listening with rapt attention. In 1979, while I was doing a medical school rotation in obstetrics at St. Thomas’s Hospital in London, Mel visited as Grand Rounds speaker. A gracious guest, she cited recent work by St. Thomas’s faculty. Always do your homework! she later said. She was also hospitable. Mel hosted Mother Teresa when the sister received an honorary degree at Harvard in 1982. Later, at Children’s Hospital (CHMC), she hosted Mildred Stahlman, who was the first to ventilate a baby. Originally competitors, Mel and Millie had become good friends.

In 1980, Mel spoke to the women MD-PhD students at HMS, recalling her father’s loving support. She shared some personal challenges, especially convalescing from TB during internship. “I had a lot of time to think about the lung – and I did!” she laughed, closing with: “don’t simply do well in your field. Create a new field. For example, we could really use a field of Information Technology!” Mel often had innovative ideas (1).

Starting my laboratory at Brigham and Women’s Hospital (BWH), seeking to learn methods of studying lung development, I collaborated with John Torday, whom Mel had brought from McGill to Harvard. When I asked for Mel’s feedback on our first manuscript (2), suggesting a meeting in her office, she replied, “Absolutely not! We’re going out to lunch!” Thus began a great friendship.

When I first presented at an American Thoracic Society meeting, Mel sat beside me, commenting, “Interesting data, but you have to change your slides from black-on-white, which doesn’t project well. Use yellow-on-blue.” Very constructive – very Mel! Writing a grant with Jackie Coalson about bronchopulmonary dysplasia (BPD), I called Mel. At lunch, she patted my arm, “Mary, BPD isn’t a problem anymore.” By the 1990s, with surfactant therapy for RDS, BPD had become milder than originally described (3). Regardless, Mel knew BPD remained a challenge, and even collaborated with Jackie herself (4).

Mel encouraged countless young professionals, especially women, but also men. Her magic stemmed from unconditional faith in others. Lewis First, then Assistant Professor and previously Mel’s intern, was asked by Mel to co-edit a new Pediatrics textbook with her (5). Lewis is now a Pediatrics Chairman (6). Mel thought highly of Mary Williams from Boston University, who had done a sabbatical in CHMC Neonatology, and often sought Mary’s valuable critique. When Stella Kourembanas became a Neonatology SCCOR Program Director at CHMC, Mel was delighted and attended monthly meetings, closely following our progress with interest. During those years, it seemed that Mel was passing the baton to Stella, one of her closest protegés. Now, Stella is Director of Newborn Medicine at CHMC.

Mel’s enthusiasm was infectious. Once she asked me to tour the new Beth Israel NICU with her. The facilities and care were impressive, yet the highlight was one infant who would only drink mother’s milk, but not from a bottle – so they tried feeding her milk from a cup. Breakthrough! Mel was excited because many developing countries have insufficient bottles. This simple success could save lives. Another day, Mel whisked me to lunch with another pediatrician who was returning to clinical work after years of disability. By dessert, we were energized, embracing the future with Mel.

When someone asked Mel how she felt about not having any children, Mel smiled, “What do you mean? I’ve had thousands of them!” There were no problems, only challenges; no regrets, only opportunities. Working with UNICEF deepened her awareness of global health needs, often simple yet unattainable. In India, she saw three babies in one NICU incubator and asked if they were triplets. No, they said, it was their only incubator. There weren’t sufficient resources to save all babies. She spread the word.

Mel rejoiced when her trainees had children. When I was 8 months pregnant with my third child, I invited Mel for dinner at our home. She brought Maine blueberry jam, happy to meet my family. My 2-year-old son delighted her by ooh-ing and ahh-ing over the dessert, hoping to skip dinner. She later sent a warm thank you note, including 20 unusual baby names she’d collected from the NICU.

In 1994, Mel was elected into the National Academy of Sciences for her discovery that newborns require surfactant to breathe (7, 8). Her comment? “Imagine that! I never even published in that journal!” Later she ran for AAAS President (9) opposite an engineer whose essay detailed his leadership experience. Mel’s essay simply addressed many challenges facing science: needs for improved rice production, vaccinations, clean water, recognizing children as our most precious resource. Mel won the election.

Mel was nobody’s fool. She would tell her new ideas to over 14 people so everyone knew the ideas were hers; then she would publish quickly. She was neither offensive nor defensive. In Japan (10), Mel said: “I close with a quotation attributed to a famous German pathologist in the last century (Virchow): all new knowledge goes through three phases: (1) it is ignored; (2) it evokes hostility; (3) haven’t we known this all along?”

Mel lived a full life. She often traveled with a companion. Her niece, Sue, wrote: “everyone in our family had trips with Mel including my folks. Moreover, she often took friends/colleagues with her on trips to various parts of the world.” CHMC Neonatology held a surprise 70th birthday party for her, with ~30 people telling Mel stories. When she received the Howland Award, Pediatric’s greatest award, her previous intern, Margaret Hostetter presented it (6). Her post-award celebration included Lewis First’s musical rendition of “Mel,” which she enjoyed singing at home. She was delighted to stop and smell the roses.

After moving to Duke in 2004, I visited Mel every year around Thanksgiving. Once, I drove her to pick up a complete turkey dinner for her family. Mel had never learned to cook, but that never stopped her from having a party. Another time at her home, Mel showed me her nametag collection from the meetings she had attended. In 2007, Mel was animated about the BBC coming to interview her.

Then, Mel became increasingly forgetful and stopped coming to CHMC. She had live-in nursing care, thanks to her family: Sue, Bill, Jennifer, and Carl Smith. At home, Mel held tightly to her biography, written by Bojan Jennings, her chemistry professor from Wheaton College (11). She was clinging to memories, precious jewels slipping away.

The last time I saw Mel was 5 days before she died, at a nursing home near her childhood home. Sue and Bill had filled her room with her awards, nametags, and family photographs: a lifetime of love and accomplishment. At that moment, all I did was sit beside her, holding her hand. She was awake and comfortable, gazing into the distance and speaking in an unknown language to someone only she could see.

On December 4, 2011, with Sue and Bill beside her, Mel became a free spirit. Her funeral was at the church she’d attended as a child. Like Mel, it was unpretentious and heartfelt, with family and a few friends Linda van Marter and I went together. Fred Lovejoy extolled her influence at CHMC. Sue spoke a universe of love. Bill introduced Mel as The Personal Physician for the Smith Family: “take 2 aspirins and call me in the morning.” How marvelous that Mel’s family has the same sense of humor!

**Figure 1:**
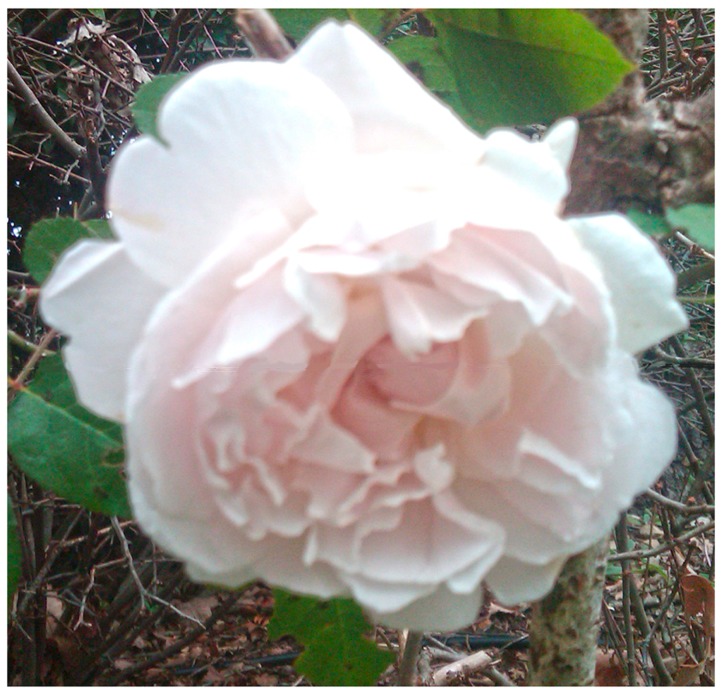
**The Rose sent by Mel**. The Redouté Rose was named after Joseph-Pierre Redouté, a Belgian artist who painted roses for Marie Antoinette, then for Josephine Bonaparte, “the rarest and most beautiful plants obtainable.” He was immortalized through his timeless inspiration of others. **Just like Mel**.

Returning home from Mel’s funeral, I gazed out at our brown December garden and a rose bush that had been dead for over a year, surprised to discover – a piece of paper? No, it was a perfect full-blown pink rose (Figure 1). “Mel did it!” I thought. She could move mountains – of course it was Mel! That single rose lasted over 3 weeks in winter weather. Mel, you were right: we only have to do one thing well. Love is the key: love of scientific discovery and humanity. Giving everything to help children, Mel transcended departmental expectations by founding neonatology. She transcended academics by making the world her institution through UNICEF. Ultimately, she transcended time by living on in the hearts of all. Mel’s greatest legacy was her inspiration of so many to believe in themselves and what they can do to make the world a better place, symbolized by a perfect rose.
